# Dataset on predictive compressive strength model for self-compacting concrete

**DOI:** 10.1016/j.dib.2018.02.008

**Published:** 2018-02-09

**Authors:** O.M. Ofuyatan, S.O. Edeki

**Affiliations:** a Department of Civil Engineering, Covenant University, Ota, Nigeria; b Department of Mathematics, Covenant University, Ota, Nigeria

**Keywords:** Predictive model, Compressive strength, Water cement ratio, Day-length

## Abstract

The determination of compressive strength is affected by many variables such as the water cement (WC) ratio, the superplasticizer (SP), the aggregate combination, and the binder combination. In this dataset article, 7, 28, and 90-day compressive strength models are derived using statistical analysis. The response surface methodology is used toinvestigate the effect of the parameters: Varying percentages of ash, cement, WC, and SP on hardened properties-compressive strengthat 7,28 and 90 days. Thelevels of independent parameters are determinedbased on preliminary experiments. The experimental values for compressive strengthat 7, 28 and 90 days and modulus of elasticity underdifferent treatment conditions are also discussed and presented.These dataset can effectively be used for modelling and prediction in concrete production settings.

**Specifications Table**

**Table:** 

Subject area	*Civil Engineering*
More specific subject area	*Production of concrete and strength properties*
Type of data	*Table, graph.*
How data was acquired	*Laboratory experiment via response surface methodology*
Data format	*Raw and Analysed*
Experimental factors	*Modelling and concrete strength*
Experimental features	*Compressive strength and self-compacting concrete*
Data source location	*Experimental and laboratory*, Nigeria
Data accessibility	*Within this article.*

**Value of the data**

• The present data can be used to predict the strength of auto-compacting concrete at varying days.
• The dataset can be used to determine the trend of strength associate with concrete.
• The dataset can be used to detect the effect of SP.
• The dataset can be used to determine the nature of concrete, and the corresponding degree of hydration.
• The dataset can serve as an experimental framework for the analysis of other basic properties of concrete.
• The dataset can help in developing experimental programme for the evaluation of model accuracy and precision.

## 1 Data, and experimental design

**Fig. 1:**
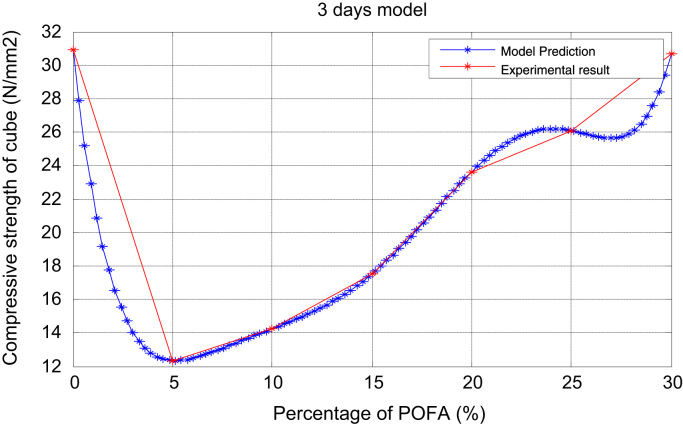
Prediction and experimental result (3-days model).

**Fig. 2:**
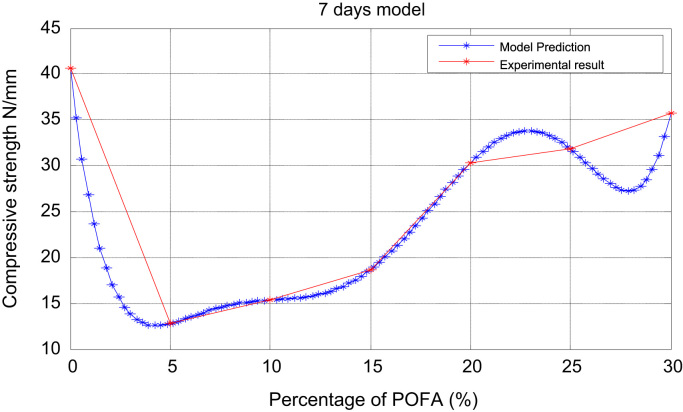
Prediction and experimental result (7-days model).

**Fig. 3:**
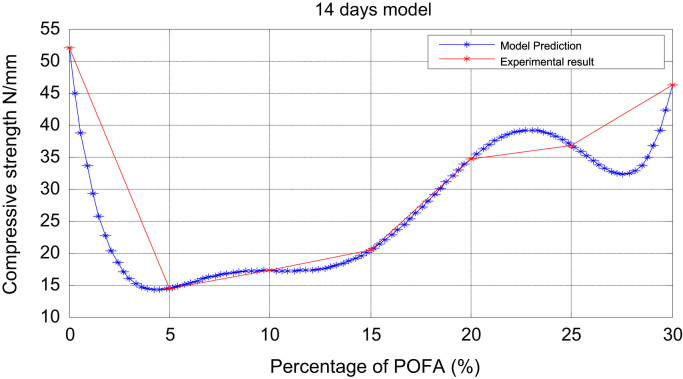
Prediction and experimental result (14-days model).

**Fig. 4:**
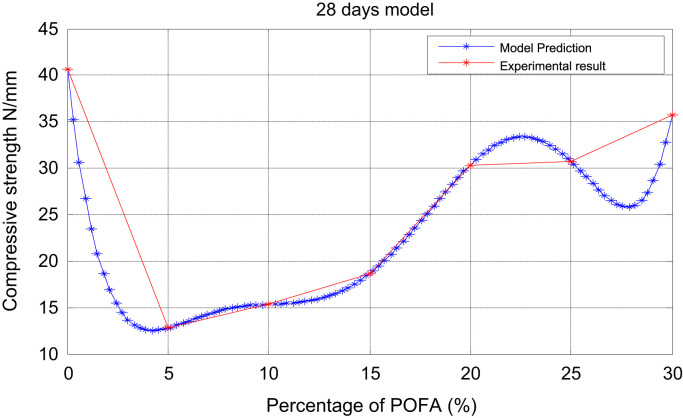
Prediction and experimental result (28-days model).

**Fig. 5:**
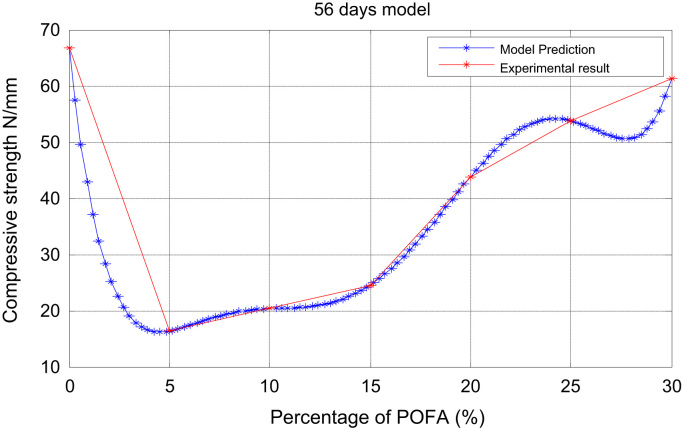
Prediction and experimental result (56-days model).

Strength data presented here are from seventy-two (72) different POFA concrete samples fabricated to compare with normal concrete without ash. We make reference to [1], [2], [3], [4], [5], [6], [7], [8] for related views such as forecasting and prediction. In this dataset article, a 7-day, 28-day, and 90-day compressive strength models were derived by statistical analysis and the proposed models results and description as contained in Table 1, Table 2, Table 3, and Fig. 1, Fig. 2, Fig. 3, Fig. 4, Fig. 5 are as follows.

**Table 1:** 3days experiment.

x	0	5	10	15	20	25	30
y	30.94	12.34	14.24	17.52	23.63	26.08	30.68

**Table 2:** 7 daysexperiment.

x	0	5	10	15	20	25	30
y	40.60	12.77	15.35	18.67	30.32	31.80	35.78

**Table 3:** ppp 14 daysexperiment.

x	0	5	10	15	20	25	30
y	52.10	14.45	17.24	20.40	34.71	36.82	46.30

### 1.1 Sample preparation methods

In this investigation, concrete samples were prepared using Palm Oil Fuel Ash (POFA) at varying percentages, with ordinary Portland cement. The POFA was replaced at 5%, 10%, 15%, 20%, 25%, and 30% with cement and superplasticer at 2%.

## 2 Materials and methods

### 2.1 Quadratic equation generated from the model

*Besides the statistical software used in the data analysis, is a predictive quadratic model defined as follows:*

(1) $$f\left(x\right)=C_{0}+\frac{C_{1}\left(x-x_{0}\right)}{1!\left(5^{1}\right)}+\frac{C_{2}\left(x-x_{0}\right)\left(x-x_{1}\right)}{2!\left(5^{2}\right)}+\frac{C_{3}\left(x-x_{0}\right)\left(x-x_{1}\right)\left(x-x_{2}\right)}{3!\left(5^{3}\right)}+\frac{C_{4}\left(x-x_{0}\right)\left(x-x_{1}\right)\left(x-x_{2}\right)\left(x-x_{3}\right)}{4!\left(5^{4}\right)}\;+\frac{C_{5}\left(x-x_{0}\right)\left(x-x_{1}\right)\left(x-x_{2}\right)\left(x-x_{3}\right)\left(x-x_{4}\right)}{5!\left(5^{5}\right)}+\frac{C_{6}\left(x-x_{0}\right)\left(x-x_{1}\right)\left(x-x_{2}\right)\left(x-x_{3}\right)\left(x-x_{4}\right)\left(x-x_{5}\right)}{6!\left(5^{6}\right)}$$

where x and $C_{i},\;i\geq 0$ denote varying percentages of POFA and compressive strength respectively.

### 2.2 Data analysis

For x the varying percentages of POFA with zero (0) as the control, andy the average compressive strength, we present in Table 1, Table 2, Table 3, Table 4, Table 5, Table 6 the relationship between xandy at varying intervals in days.

**Table 4:** 28 daysexperiment.

x	0	5	10	15	20	25	30
y	40.60	15.72	17.85	22.63	38.35	48.70	55.74

**Table 5:** 56 daysexperiment.

x	0	5	10	15	20	25	30
y	66.80	16.35	20.36	24.56	43.80	53.79	61.32

**Table 6:** 90 daysexperiment.

x	0	5	10	15	20	25	30
y	80.50	17.28	21.40	26.77	57.09	59.54	75.60

It is noted from Table 1, Table 2, Table 3, Table 4, Table 5, Table 6 that there was an increase in strength as the percentage of POFA increased but the control was slightly high. Table 7 shows the experimental and numerical results for POFA with regards to Compressive Strength for 7, 28, and 90 days.

**Table 7:** The experimental and numerical results.

	7 days		28days		90days	
POFA (%)	Compressive Strength (N/mm^2^)		Compressive Strength (N/mm^2^)		Compressive Strength (N/mm^2^)	
	Experimental	Numerical	Experimental	Numerical	Experimental	Numerical
0	40.6	40.6	59.6	59.6	80.5	80.5
5	12.77	12.77	15.28	15.28	17.28	17.28
10	15.35	15.35	19.41	19.41	21.40	21.40
15	18.67	18.67	22.47	22.47000005	26.77	26.77
20	30.32	30.32	38.10	38.100000020	57.09	57.09
25	31.8	31.800000305	41.5	41.5100050125	59.54	59.54000001
30	35.78	35.48001000125	53.48	53.4800100125	75.60	75.6000000625

The plots of the experimental values of the compressive strength at varying days vs the predicted strength using Matlab are shown via Fig. 1 through Fig. 5. The compressive strength values of POFA concrete were very close to the strength of normal concrete: 75.60 N/mm^2^, 80.5 N/mm^2^ respectively at 90 days.

### 2.3 Models correlation – predicted and measured

Matlab statistical software was used to analyse and investigate the effect of the parameters (cement, water cement (WC) ratio, POFA and superplasticiser (SP) on the hardened properties (compressive strength at 7, 28 and 90 days. Determination of the independent parameters with respect to their percentage replacement was made on initial experiments as shown in Table 1, Table 2, Table 3, Table 4, Table 5, Table 6, which also contains the experimental values of the increase in strength at 7, 28 and 90 days. The quadratic model equation was used to determine the experimental values and compared with the model. The predictive and experimental models showed the same values and pattern graphically as depicted in Fig. 1, Fig. 2, Fig. 3, Fig. 4, Fig. 5.
